# Transient response to high‐dose niacin therapy in a patient with NAXE deficiency

**DOI:** 10.1002/jmd2.12425

**Published:** 2024-05-09

**Authors:** Fatema Al‐Amrani, Khalid Al‐Thihli, Eiman Al‐Ajmi, Amna Al‐Futaisi, Fathiya Al‐Murshedi

**Affiliations:** ^1^ Pediatric Neurology Unit, Department of Child Health Sultan Qaboos University Hospital, Sultan Qaboos University Muscat Sultanate of Oman; ^2^ Genetic and Developmental Medicine Clinic, Department of Genetics Sultan Qaboos University Hospital, Sultan Qaboos University Muscat Sultanate of Oman; ^3^ Department of Radiology and Molecular Imaging Sultan Qaboos University Hospital, Sultan Qaboos University Muscat Sultanate of Oman; ^4^ Department of Child Health College of Medicine and Health Sciences, Sultan Qaboos University Muscat Sultanate of Oman

**Keywords:** COX2 inhibitors, early onset progressive encephalopathy with brain edema and/or leukoencephalopathy −1 (PEBEL‐1), NAXD‐encephalopathy, NAXE‐encephalopathy, niacin, PEBEL‐2, vitamin B3

## Abstract

**Background:**

NAXE‐encephalopathy or early‐onset progressive encephalopathy with brain edema and/or leukoencephalopathy‐1 (PEBEL‐1) and NAXD‐encephalopathy (PEBEL‐2) have been described recently as mitochondrial disorders causing psychomotor regression, hypotonia, ataxia, quadriparesis, ophthalmoparesis, respiratory insufficiency, encephalopathy, and seizures with the onset being usually within the first three years of life. It usually leads to rapid disease progression and death in early childhood. Anecdotal reports suggest that niacin, through its role in nicotinamide adenine dinucleotinde (NAD) de novo synthesis, corrects biochemical derangement, and slows down disease progression. Reports so far have supported this observation.

**Methods:**

We describe a patient with a confirmed PEBEL‐1 diagnosis and report his clinical response to niacin therapy. Moreover, we systematically searched the literature for PEBEL‐1 and PEBEL‐2 patients treated with niacin and details about response to treatment and clinical data were reviewed. Furthermore, we are describing off‐label use of a COX2 inhibitor to treat niacin‐related urticaria in NAXE‐encephalopathy.

**Results:**

So far, seven patients with PEBEL‐1 and PEBEL‐2 treated with niacin were reported, and all patients showed a good response for therapy or stabilization of symptoms. We report a patient exhibiting PEBEL‐1 with an unfavorable outcome despite showing initial stabilization and receiving the highest dose of niacin reported to date. Niacin therapy failed to halt disease progression or attain stabilization of the disease in this patient.

**Conclusion:**

Despite previous positive results for niacin supplementation in patients with PEBEL‐1 and PEBEL‐2, this is the first report of a patient with PEBEL‐1 who deteriorated to fatal outcome despite being started on the highest dose of niacin therapy reported to date.

**Synopsis**

Treatment with niacin in PEBEL‐1 did not stop disease porgression and fatality. Treatment with a COX‐inhibitor improved niacin‐related urticaria in a ptaient with PEBEL‐1.

## 1 INTRODUCTION

NAXE encephalopathy or early‐onset progressive encephalopathy with brain edema and /or leukoencephalopathy (PEBEL‐1) and NAXD encephalopathy (PEBEL‐2) are a group of autosomal recessive disorders caused by biallelic pathogenic variant(s) in *NAXE* and *NAXD* genes, respectively.^1^, ^2^, ^3^, ^4^ Biallelic pathogenic variants in *NAXE* and *NAXD* genes result in accumulation of toxic metabolites leading to mitochondrial dysfunction and enzyme inhibition.^1^, ^5^, ^6^ PEBEL‐1 and PEBEL‐2 are neurodegenerative mitochondrial disorders marked by early normal development followed by catastrophic neurovegetative regression, and sometimes cutaneous signs that tend to be triggered by febrile illnesses. Neuroregression can manifest as ataxia, hypotonia, seizures, encephalopathy, respiratory insufficiency evolving to coma and death before the third birthdate.^1^, ^2^, ^3^, ^7^, ^8^, ^9^ However, late‐onset disease with slow progression and longer survival to the 2nd and 3rd decade has been documented in the literature.^2^, ^3^

Niacin (vitamin B3) is the precursor of the functional cofactors NADH and NADPH as well as their oxidized counterparts NAD+ and NADP+.^10^ Therefore, niacin administration has been shown to pause clinical deterioration of patients with PEBEL‐1 and PEBEL‐2 and provide protection against the fatal outcome of these disorders.^2^, ^3^, ^4^, ^8^, ^11^, ^12^ In fact, no patient treated with niacin has succumbed to death as anticipated by the natural history of the disease. The longest follow‐up period described so far is 7 years. Herein, we report a 14‐month‐old male with PEBEL‐1 who was started on niacin early on his disease course. However, he continued to have recurrent episodes of encephalopathy with the disease progressing despite dose escalation resulting in his death two months after the initial presentation. The dose escalation was associated with generalized urticarial skin lesions potentially impeding compliance. We hereby also report re‐purposed use of celecoxib to mitigate the impact of niacin associated urticaria in patients with PEBEL‐1.

## 2 MATERIALS AND METHODS

### 2.1 Clinical Evaluation

The patient was assessed in a tertiary care center and assessed by multiple specialties including biochemical genetic team and neurology team. He was admitted several times to the hospital due to relapses of his disease.

### 2.2 Systematic Literature Review

A search for patients from all age groups with PEBEL‐1 or PEBEL‐2 who were treated with niacin from English publications was performed in PubMed for the last 10 years from 2013 to 2024. The following medical subject heading (MeSH) keywords were used: niacin, vitamin B3, NAXE‐encephalopathy, NAXD‐encephalopathy, PEBEL‐1 and PEBEL‐2. A thorough detailed review for these articles was performed to identify patients who were treated with niacin. Furthermore, after identification of patients who were treated with niacin, pertinent information concerning age of the patient at diagnosis, *NAXE/NAXD* variants, clinical presentation, neuroimaging findings, niacin dose, age at treatment, other treatment received, outcome, and follow‐up duration was reviewed.

A total of 19 articles were found concerning PEBEL‐1 and PEBEL‐2 describing a total of 45 patients.^2^, ^11^, ^12^, ^13^, ^14^, ^15^, ^16^, ^17^, ^18^, ^19^ 32 patients were found to have PEBEL‐1 and 13 patients were found to have PEBEL‐2. Eight patients were treated with niacin from the two groups.^2^, ^3^, ^4^, ^8^, ^11^, ^12^, ^13^ Among those, five had PEBEL‐1 and three had PEBEL‐2 (Table 1).

**TABLE 1:** Summary of *NAXE/NAXD* patients treated with Niacin.

Patient #	Author	Age at onset of symptoms / gender	Symptoms at presentation	NAXE/NAXD variant	Niacin dose (mg)/day), age	Other treatment received	Reported effect of the treatment on the patient/ age	Outcome/ duration of FU
1	Trinh et al^3^	22 yrs/F	Initially headache Respiratory insufficiency with fluctuating disease course Developmental impairment Seizures and myoclonic jerks Comatose Cerebellar ataxia Spastic quadriplegia Dysarthria Dysphagia Cervical dystonia Aseptic fever Neuropsychiatric symptoms Bulbar symptoms Hypomimia **MRI:** was unremarkable.	*NAXE*, cpd het; Splicing c.665‐1G > A and c.757G > A (p.Gly253Ser)	Initially 40 mg increased to 80 mg/day mg/kg (NA)	Co‐enzyme Q10 ASM CLB, LVT, lamotrigine, piracetam and V D3	Wheelchair bound Improved cognitive function, attention, speech, mobility, andeating Significant improvement in spasticity without any intervention apart from niacin/22 yrs	7‐year disease duration Patient was alive at publication time She could use mechanic wheelchair by her own. OE; Perioral dyskinesias, reduced visual acuity bilaterally, divergent strabismus and very mild limb spasticity with movement restriction due to fixed contractures. Leg pain treated as neuropathic pain Tpx was tapered and re‐introduced due to appearance of head tremor and back pain
2	Manor et al^2^	2 yrs/F	Ataxia Intermittent esotropia Motor regression Encephalopathy Ventilated and mechanically ventilated **MRI:** bilateral symmetrical faint hyperintense T2 signal in the superolateral aspect of the caudate nuclei.	*NAXE*, cpd het; c.804‐807delinsA (p.Lys270del) and c.368A > T (p.Asp123Val)	200/day (mg/kg) NA/2 yrs	NA	Removed from the ventilator Discharged from the hospital Muscle strength 4/5 2 yrs	Alive at 3 years with residual ataxia No crisis recurrence while on niacin derivatives Admitted with febrile illness/1 yr
3	Dan Yu et al^8^	2 yrs/F	Walking instability Limb weakness Strabismus Ataxia Decrease muscle tone Delayed development Repeated respiratory failure	*NAXE*, cpd het; c.255A > T (p.Glu85Asp) and c.361G > A (p.Gly121Arg)	NA	NA	NA	NA
4	Li‐Wei Chiu et al^4^	20 m/F	Progressive unsteady gait and hand tremor associated with febrile illness She rapidly progressed to Coma and respiratory failure/20 m (recovered) **3.6 yrs:** progressive ataxia with anisocoria **MRI:** bilateral hyperintensity in the middle cerebellar pendules in DWI. Subsequent MRI after further deterioration showed complete resolution of the diffusion signal abnormalities in the middle cerebellar peduncles, but there were T2/DWI signal abnormalities in the cerebellar hemispheres, superior cerebellar pendules, and posterior part of the brainstem. There was cerebellar atrophy in MRI done during another deterioration, happened 2 years after her initial presentation.	*NAXE*, homo; c.733A > C (p. Lys245 Gln)	30 (Nicotinamide)/mg/kg NA 3.6 yrs for 14 D	Thiamine Riboflavin Pyridoxine	Extubated after admission with acute respiratory failure/3.6 yrs	Alive at 5.5 year when article was published She could walk with unsteady gait for short distances She could communicate with short sentences and dysarthric voice She could eat with spoon Power in the 4 limbs was 4 IQ was 75 She could go to school and participate in activities under moderate supervision (1.9 yrs from niacin initiation)
5	Manor et al^12^	2 yrs/ M	2 yrs: Acute onset gait abnormalities and respiratory failure (recovered slowly to baseline but developed progressive muscle weakness) 13 yrs: evaluation for muscle weakness (no Dx found) 16 yrs: Myoencephalopathy; tinnitus, dizziness, diplopia, dysphagia, and weakness with high CPK up to 16 000 triggered by febrile illness. Respiratory failure required NIV Encephalopathy **MRI:** volume loss mainly in the cerebellar hemispheres, bilateral T2 hyperintensity in the medial aspect of the thalami. Follow‐up spinal **MRI:** showed new T2 hyperintensity in the central gray matter of the cord at C2.	*NAXD*, 8 kb microdeletion within chromosome 13q34, encompassing exons 1–2 and a homo c.46G > A (p.Val16Ile)	100 mg/day mg/kg(NA)/16 yrs	No additional supplements	11 months post niacin initiation Fully oriented, participated in age‐appropriate physical activity, decrease in CPK level to normal/17 yrs	Alive at 17 yrs (17 m)
6	Zhou et al^11^	2.8 yrs/M	Repeated episodes of: Dystonia, Neuroregression: with difficulties in standing or sitting, swallowing, or speaking + skin lesions 3.8 yrs: unsteadiness, skin lesions, neuroregression with inability to sit or stand, follow an object, swallow or speak, persistent dystonia during wakefulness Initial **MRI:** mild edema in the cerebellar hemispheres. Subsequent MRI showed bilateral T2 hyperintensity and diffusion restriction in the basal ganglia and edema in the cerebellar hemispheres bilaterally. Spinal MRI: normal. Clinical improvement was accompanied by resolution of the signal abnormalities in the brain and atrophy was noted in follow up.	*NAXD*, cpd het; c.101_102delTA (p.Thr35Phefs*63) and c.318C > G (p.Ile106Met)	500 mg/day (mg/kg) NA At 4.5 yrs (3rd episode)	**1st episode**: biotin 10, vitamin B1 vitamin B2, vitamin C, vitamin E 10, CoQ10, idebenone and carnitine He recovered to baseline and skin lesions improved without scarring (No B3 was given) 2nd episode: trihexyphenidyl, levodopa, and nitrazepam for dystonia (improved after D/C)	After the first episode was completely back to his baseline and full resolution of skin lesions without scarring and no dermatological side effects Improvement of his brain MRI After niacin (4.5 yrs); clinical symptoms stabilize	Alive at 5 yrs Skin lesions improved significantly. Neurological symptoms stabilized with no further deterioration in clinical status MRI‐brain: relief of previous brain lesions with brain atrophy 6 m from niacin initiation
7	Van Bergen et al^14^	32 yrs/M	Mild head trauma followed by rapidly worsening cognitive and behavioral disturbances multidirectional nystagmus Ataxia progressive myoclonus affecting the face and the upper limbs, autonomic dysfunction, and severe oral mucositis. Initial **MRI**: moderate cerebellar atrophy, which was present 10 years prior to that. A repeat MRI five weeks later showed multiple bilateral areas of cortical FLAIR hyperintensity and diffusion restriction. A follow‐up MRI 3 and 1/2 months later showed almost complete resolution of cortical signal abnormalities with moderate global cerebral atrophy.	*NAXD*, homo splicing; c.441 + 3A > G:p.?	500 mg/day tapered to 250 mg/day after ICU course	Coenzyme Q10, biotin, L‐carnitine and L‐arginine	Improvement in oral ulceration and encephalopathy, continued to have myoclonus MRI‐brain: resolution of cortical signal intensities with global cerebral atrophy	Months He lived for moths with tracheostomy, NG‐tube, feeding on ASM and with no change in encephalopathy No report of death
8	Almudhary et al^20^	24 yrs/M	A 24‐year‐old man first became symptomatic in infancy with frequent initial neurological decompensations in the setting of infections with subsequent clinical improvement followed by stability at the age of 2.5 yrs with residual cerebellar dysfunction. **MRI:** Cerebellar and spinal cord atrophy.	*NAXE*, homo; c.733A > C, (p.Lys245Gln)	Additional niacin 40 mg twice daily was added at the age of 19 yrs	L‐Carnitine 330 mg once daily, coenzyme Q10 200 mg twice daily, B‐100 complex once daily (daily (100 mg of vitamins B1, B2, B3, B5 (pantothenic acid), and inositol, 10 mg of bioactive vitamin B6, 1000 mcg of folic acid, 10 mg of choline, 500 mcg of bioactive vitamin B12, and 300 mcg of biotin in each tablet), and riboflavin 200 mg once daily at the age of 2.5 yrs	Improvement and prevention of acute episodes after the age of 2.5 years with starting mitochondrial cocktail (contains already 100 mg of Niacin)	Alive at 24 yrs, clinical features noted over the years include chronic ataxia, nystagmus, ptosis, mild spasticity of lower limbs, and neuropsychiatric symptoms.
8	Our patient	14 m/ M	Encephalopathy (lost interest in playing, reduced activity and reduced oral intake) 1 month later: episodic head drop **MRI:** bilateral symmetric T2/FLAIR hyperintensity and increased signal in DWI in the middle cerebellar peduncles and diffuse myelopathy. Follow‐up MRI showed significant improvement of the signal abnormalities in the brain and spinal cord despite the clinical deterioration.	NAXE, homo; c.827del p.(Pro276Hisfs*43).	Initially 200 mg Increased until reached 600 mg/day 14 m	Co‐enzyme Q Levocarnitine Biotin Pyridoxine Thiamine	Patient had improvement after each increase in niacin dose. Effect lasted for few days to weeks mainly with improvement in encephalopathy However, patient continued to deteriorate despite increasing niacin dose/15 m	Death at 16 m

Abbreviations: ASM, anti‐seizure medication; CLB, clobazam; cpd, compound; D/C, discharge; Dx, diagnosis, DWI, diffusion‐weighted imaging; F, female; het, heterozygous; LVT, levetiracetam; M, male; m, months; MRI, magnetic resonance imaging; NA, not available; NG‐tube, nasogastric tube; NIV, non‐invasive ventilation; OE, on examination; Tpx, topiramatehomo, homozygous; V, vitamin; yrs, years.

## 3 RESULTS

### 3.1 Clinical report

A male child with an uneventful perinatal history and normal initial development presented at 14 months of age with encephalopathy (reduced activity, irritability, and loss of interest in exploring toys), ataxia, and intermittent episodes of head and trunk titubation of one month duration. The child was born to consanguineous parents and had two affected siblings with PEBEL‐1 previously confirmed to have homozygous c.827del p.(Pro276Hisfs*43) in *NAXE*. The older sibling of the index patient had an uneventful perinatal history and normal early development. She presented with intermittent torticollis after 6‐month's vaccination. She never achieved walking. At 18 months, she developed retrogression, lost head control, developed eye misalignment, and encephalopathy. MRI‐brain showed marked edema and diffusion restriction in the cerebellar and brainstem. She was intubated and mechanically ventilated and passed away at 19 months of age. The second affected sister had an uneventful perinatal history and normal initial development until the age of 18 months when she developed intermittent anterocollis, lost her ability to walk, and became ataxic after 18‐month's vaccination. Two weeks thereafter, she had sudden deterioration with severe axial hypotonia, respiratory failure, ophthalmoplegia and recurrent seizures that required intubation and mechanical ventilation. She passed away within a week from the history of deterioration. The diagnosis was made after the death of the second sister, so neither of the index patient's affected sisters was started on Niacin.

**FIGURE 1:**
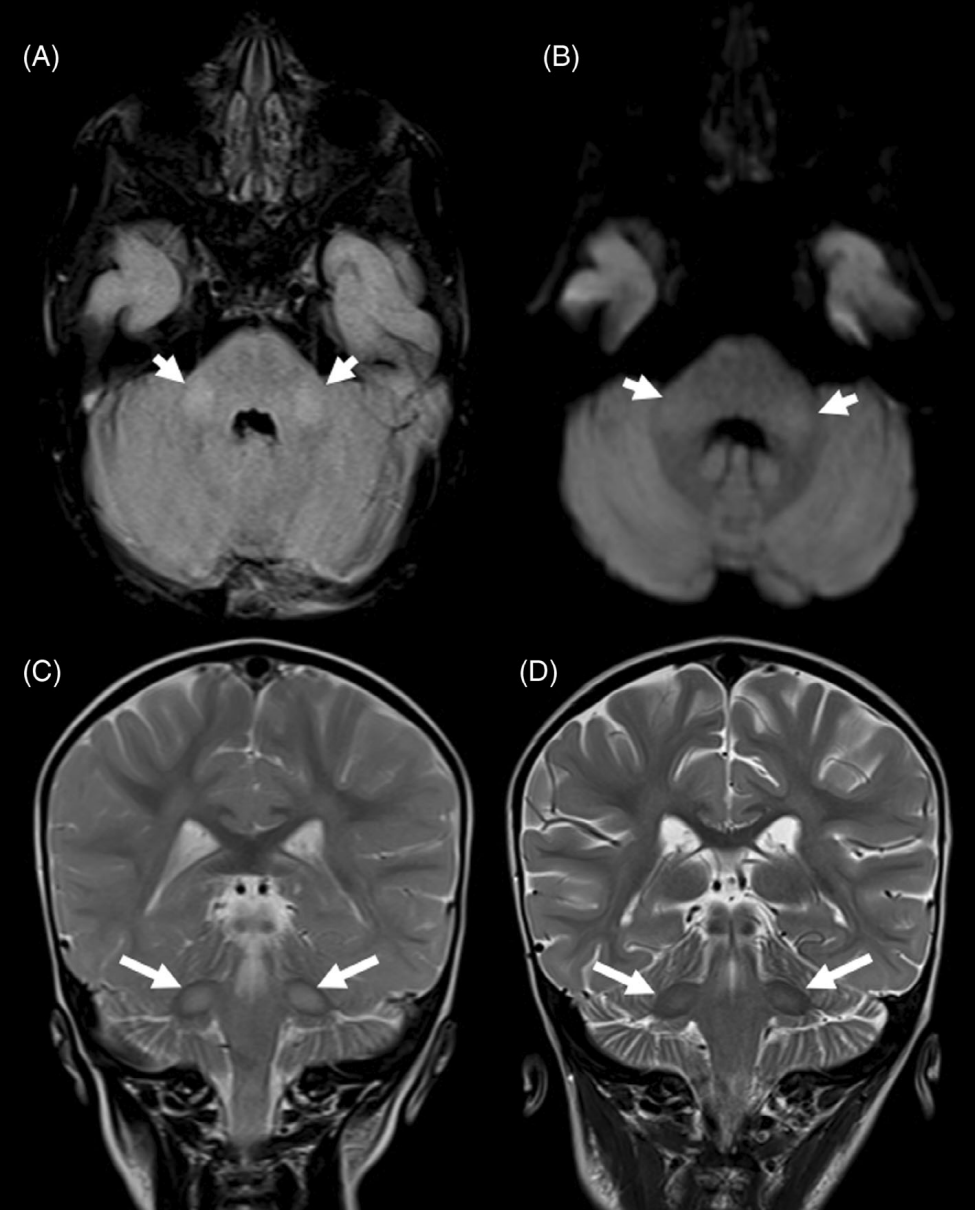
MRI of the brain before and after treatment. (A) Axial FLAIR image shows bilateral faint hyperintensities in the middle cerebellar peduncles (arrows). (B) The signal abnormalities show a faintly increased signal on diffusion‐weighted image (arrows) without diffusion restriction (the ADC map is not shown). (C) Coronal T2‐weighted image demonstrates the signal abnormality in the middle cerebellar peduncles before the treatment with vitamin B complex (arrows). (D) Interval radiological improvement of the signal abnormality in middle cerebellar pendules after treatment (arrows). Mild brain atrophy is also present.

**FIGURE 2:**
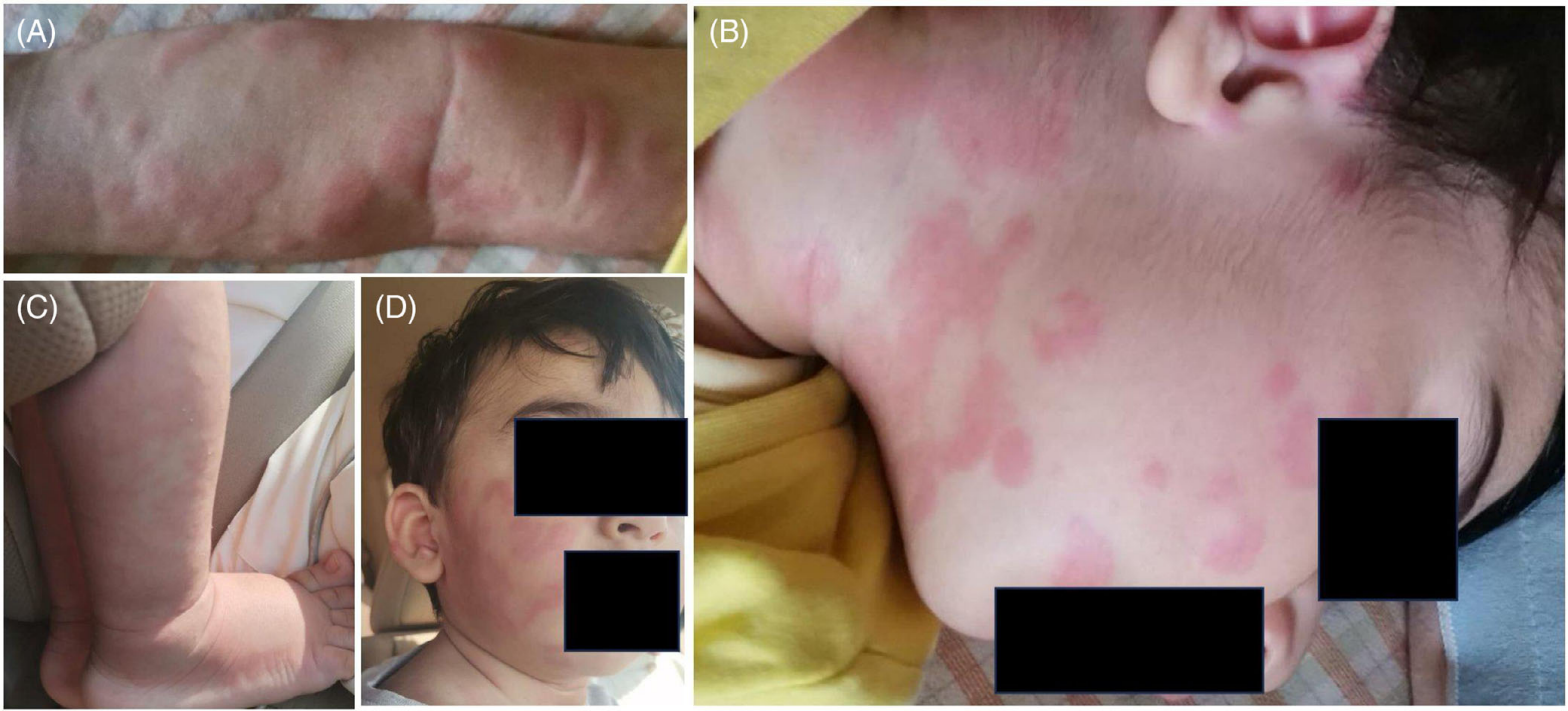
(A–D) Showing the urticarial rash attributed to niacin.

**FIGURE 3:**
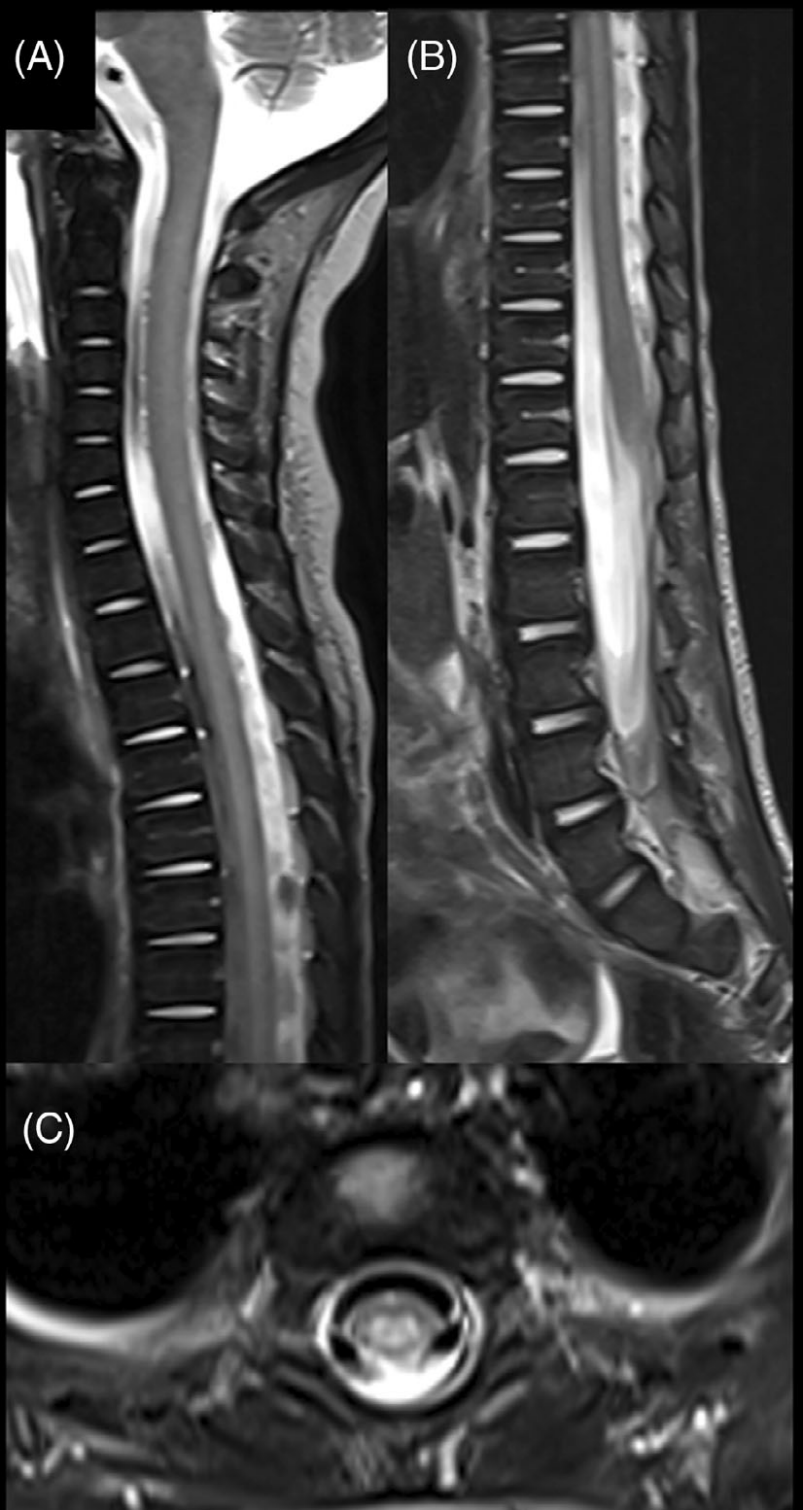
MRI of the spine after treatment. (A, B) Sagittal T2‐weighted images demonstrate abnormal T2 hyperintensity involving nearly the entire spinal cord. (C) Axial T2‐weighted image shows the central distribution of the signal abnormality in the spinal cord with relative sparing of the periphery of the cord.

The index patient was tested for the known familial variant and was confirmed to be homozygous. Brain MRI performed at 14 months (4 weeks from symptom onset) showed symmetric T2/FLAIR hyperintensity in the middle cerebellar peduncles bilaterally (Figure 1A–C). He also had diffuse myelopathy without abnormal enhancement. Investigations including plasma lactate, ammonia, creatinine phosphokinase (CPK), liver function test (LFT), blood gas, and carnitine (free and total) were all within normal limits. Niacin was introduced at a dose of 50 mg once daily and increased by 50 mg per day reaching 200 mg once daily (20 mg/kg/day) in addition to vitamin B complex, thiamine and pyridoxine. The patient responded positively regaining ability to walk independently and normal activity and cognition within two days from therapy initiation. The patient, however, developed an urticarial rash attributed to niacin and was initially managed with antihistamines (Figure 2). Despite initial stabilization on niacin, there was a concern about the persistence of urticarial rash. Given the apparent improvement niacin was believed to have brought, clinical judgment called on continuation of niacin despite the cutaneous complications. In addition to antihistamines when necessary, celecoxib 50 mg twice daily (10.5 mg/kg/day) was added based on the previously documented benefit of COX2 inhibitors inducing niacin‐tolerance in adult patients, which resulted in remarkable improvement in his urticaria. Despite compliance with niacin, he presented with excessive irritability, lethargy progressive neuroregression with loss of motor and communication skills seven weeks after his initial presentation. Niacin was increased then to 250 mg once daily. However, he presented three days later with reduced level of consciousness, neck rigidity, and persistent high‐grade fever. He transiently improved after admission and regained his head control and effective communication. However, he rapidly deteriorated thereafter with irregular shallow breathing, hypotension, and hypothermia. He was intubated and shifted to pediatric intensive care unit (PICU) nine weeks after his initial presentation. Niacin dose was escalated gradually by 50 mg per day to 500 mg once daily and subsequently to 300 mg twice daily. Throughout his intensive care unit course and despite being on 600 mg daily, he continued to be severely encephalopathic with no focusing and no response to the surroundings. He continued to have intermittent hypotension, episodes of severe bradycardia, and hypothermia that tended to settle temporarily with each increment in niacin dose. When he was on niacin at a dose of 600 mg/day, the bradycardias were less frequent, and his blood pressure was maintained. On examination then, he had neck stiffness, he opened his eyes spontaneously, moved all 4 limbs and grimaced to pain. Pupils were pinpoint with sluggish reaction to light. He had generalized hypertonia predominantly in the lower extremities with brisk deep tendon reflexes and clonus on both sides. MRI was performed again and revealed significant improvement of the signal abnormality in the middle cerebellar peduncles and the spinal cord despite the clinical deterioration (Figure 1D, Figure 3). An attempt to extubate the patient in the PICU failed as he was completely apneic with closed vocal cords, and he was immediately reintubated. At this point, the treating team felt further escalation of niacin is unlikely to result in any meaningful recovery or positive effect on his quality of life. Therefore, the decision was made to stop niacin and move toward comfort care. Despite PICU care, the patient continued to experience multiple episodes of desaturation and bradycardia and died three days after discontinuing niacin.

## 4 DISCUSSION

Niacin was used in previously reported patients as a therapeutic option that might ameliorate the PEBEL‐1 and PEBEL‐2 symptoms (Table 1).^2^, ^3^, ^4^, ^9^, ^11^, ^12^, ^13^, ^15^ Our patient received the largest niacin dose reported for PEBEL‐1 treatment up to 600 mg per day.^2^, ^3^, ^4^, ^9^, ^11^, ^12^, ^13^, ^15^ There was clear transient improvement in the encephalopathic state and neurological symptoms with each dose increment. Despite the fact that he decompensated shortly after each dose increment, transient improvement was clearly evident. He passed away at the age of 16 months despite being given niacin within 4 weeks of the onset of symptoms.

Trihn et al proposed niacin as a therapeutic option for PEBEL‐1.^3^ That was based on the observation of *NAXE* knock‐out human cells that showed higher levels of accumulated NADHX compared to wild‐type cells and subsequently NAD+ depletion.^2^ In the same study, authors raised the question of the possible underlying pathophysiological mechanisms in PEBEL‐1 where two possible pathophysiological mechanisms were proposed. One was the possibility of reduced functional pools of NAD(P) cofactors. The second pathophysiological mechanism was due to the toxic effect of accumulated NAD(P)HX metabolites.^2^

**FIGURE 4:**
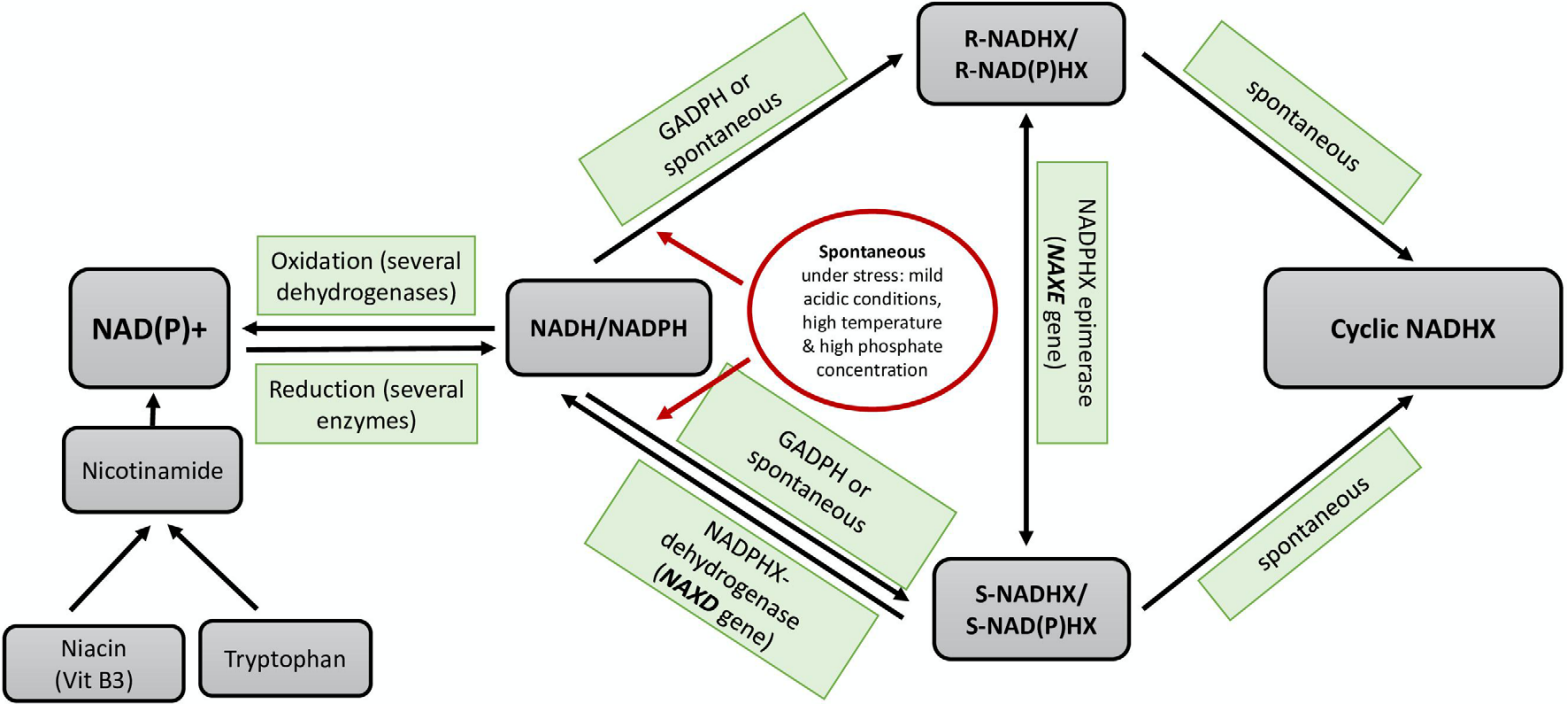
Schematic diagram showing NAD(P)+/NAD(P)H metabolism and repair system and the relation with vitamin B3 (niacin). NAXE or NAXD deficiency leads to increased formation of NAD(P)HX (R, S, or cyclic forms) which depletes NAD(P)H pool and encompasses additional toxicity by inhibiting several dehydrogenases. Vitamin B3 supplementation was hypothesized to prevent acute depletion of NAD(P)H during inflammatory crises in patients with NAXE and NAXD deficiency (adapted from Kremer.et.al & Chiu LW. et.al). NAD: nicotinamide adenine dinucleotide (reduced form, NADH; oxidized form, NAD+), NADP: nicotinamide adenine dinucleotide phosphate (reduced form, NADPH; oxidized form, NADP+), R‐NAD(P)HX, S‐NAD(P)HX and cyclic‐NAD(P)HX: hydrated metabolites of NAD(P)H, GADPH: glyceraldehyde‐3‐phosphate dehydrogenase, NAXD: NAD(P)HX dehydratase, NAXE: NAD(P)HX epimerase.

The idea of supplementation with NAD precursors including niacin was proposed to help overcome NAD(P)HX repair deficiency and therefore decrease the risk of decompensation with the trigger(s) in the future^2^ (Figure 4). Manor et.al speculated that daily niacin supplementation prevents acute depletion of NAD(P)H during inflammatory crises. However, the authors suggested further studies to support this hypothesis of possible chronic low‐grade of depletion of NAD+ pool.^2^ Moreover, nicotinate and nicotinamide metabolites were either absent or reduced when tested in one patient along with elevation in branched chain amino acid degradation metabolites. These abnormalities normalized when checked six months after daily niacin supplementation from the acute crises.^2^

Trihn et al reported one patient who had a response to niacin. She was treated with a 40 mg once daily dose, and subsequently, the dose was increased to 40 mg two times daily. The author reported that the patient was still wheelchair bound, but she showed improvement in her cognitive function, attention, speech, mobility, and eating. Furthermore, the author reported significant improvement in spasticity post‐niacin supplementation without any intervention (Table 1).^3^ Moreover, Manor et al reported a 2‐year‐old patient presented with ataxia, esotropia, regression in gross motor skills, and encephalopathy. The patient was given 200 mg of niacin supplementation per day after the establishment of her diagnosis. The patient improved and she was alive at the time of publication of the author's article with only residual ataxia (Table 1).^2^ In addition, Li‐We Chiu reported a 20‐month‐old patient who presented with ataxia and hand tremors in the context of mild febrile illness. However, she rapidly progressed to coma, respiratory insufficiency, and quadriplegia. Shortly after, she was started on nicotinamide and reported to have an improvement in her quadriparesis and her cognitive function **(**Table 1 **)**.^4^ However, this patient received nicotinamide for 14 days only and she continued to show improvement after discharge while on rehabilitation.

Additionally, niacin therapy improved the clinical conditions of two patients with NAXD‐deficiency (PEBEL‐2) (Table 1).^11^, ^15^

The limited number of patients reported to date supports that niacin has a positive impact in preventing further severe metabolic crisis or decompensations. All niacin‐treated patients reported in the literature had no crises after initiation of niacin, and all niacin‐treated patients reported so far were alive and stable at the time of published articles albeit with residual neurological deficits.

However, it is still premature to attribute all previously observed improvement/stabilization to niacin treatment alone keeping in mind the possible publication bias driving publication of positive effect of therapy despite the short follow‐up periods.

PEBEL‐1 and PEBEL‐2 are progressive and lethal disorders, resulting in death in early life.^1^, ^2^, ^5^ Among the 23 patients reported, all passed away in the first decade of life shortly after their initial presentation (vast majority in the first 2 years of life), apart from a few exceptions.^1^, ^2^ Only seven patients reported to be alive and four of them survived until the third decade.^2–4,11–13,15^ Those who survived until the third decade had a late age of disease onset except for the one patient reported by Almudhary et al.^3^, ^12^, ^13^ This patient, currently 24‐years‐old, was symptomatic in infancy (15 months) but is still alive and stable despite residual ocular and cerebellar signs. He improved and relatively stabilized after starting mitochondrial vitamins (that included 100 mg daily of niacin) at the age of 2.5 years.^13^

Furthermore, a handful of patients had acute episodic deterioration, which seemed self‐limited, with spontaneous recovery without or before receiving niacin.^4^, ^11^, ^15^ Trinh et al. reported 22‐year‐old with PEBEL‐1 who recovered from her episode and tracheostomy was removed before niacin initiation.^3^ Additionally, Li‐Wei Chiu et al. a reported 20‐month‐old female presented with unsteady gait rapidly progressed to coma and respiratory failure. She improved to independent walking without niacin. She presented two years later with acute deterioration and niacin was initiated for 14 days only.^4^ Manor et al. reported a patient with PEBEL‐2 presented initially at the age of 2 years and then 13 years, he recovered spontaneously in both episodes. He received niacin at the age of 16 years after confirmation of molecular diagnosis.^11^ Finally, Zhou et al. reported a patient with PEBEL‐2 presented at the age of 2 and 3 and 10 months. He recovered spontaneously to baseline in both episodes without Vit B3. Vit B3 supplementation was started after the third episode at 4 years and 5 months.^15^

The patient we report, however, continued to deteriorate and the apparent improvement following introduction of niacin was rather transient. The transient improvement initially seen with niacin prompted consideration of dose increment in the hope that the sustained response might be dose dependent, particularly when the patient deteriorated despite being on a dose of 250 mg per day. Although the patient reached a total dose of 600 mg/day of niacin, he continued to be encephalopathic and when signs of impending brainstem dysfunction were seen, niacin therapy was withdrawn, and death followed thereafter. We hence argue that niacin has failed in halting progression or attaining stabilization of the disease in our patient despite being subjected to the highest dose of niacin reported to date in these patients.^2^, ^3^, ^4^, ^9^, ^11^, ^12^, ^13^, ^15^

The transient improvement this patient showed despite the severe null mutation argues for the benefit niacin provides as a precursor of NAD independent of the NAXE activity. This also raises the question about whether such a response may be sustained in patients with severe null mutations as previous reported patients with favorable response to niacin had missense variant(s) either in one copy or both copies (Table 1). There is no current report of response to niacin (positive or negative) in a patient with biallelic truncating NAXE variants.

The failure of niacin therapy in attaining sustained improvement raises as well the question about whether previously published patients with apparent improvement remain at risk of catastrophic life‐threatening encephalopathy, a very important contribution to counseling of patients started on therapy and a call for longitudinal follow‐up studies or global patients' registries to monitor patients on therapy. Moreover, there is genuine concern about the optimal niacin dose given the hypothetical concern that excess NAD(P) may give rise to the hydrated toxic isoforms that can keep accumulating in NAXE and NAXD deficiencies. Presymptomatic treatment was not described as well, an approach that may potentially has an added value to the expected response to Niacin treatment.

Another clinically relevant observation in our patient was the development of severe urticarial rash as an adverse effect to niacin. This niacin adverse event has not been reported previously in patients with PEBEL‐1 treated with niacin.^2^, ^3^, ^4^ However, it has been reported in patients receiving niacin for other indications.^21^ The first reported patient with such adverse event has been reported in 1938. Subsequent case reports followed.^21^ Our patient had the dermatological adverse event at a low dose of 100 mg once daily and his symptoms got worse with dose escalation. COX2 inhibitors were reported to induce niacin tolerance in patients receiving niacin for other indications.^20^ The use of celecoxib and aspirin helped treat patient's symptoms and enabled continuation of therapy. COX2 inhibitors reduce cutaneous side effects in patients taking niacin therapy through inhibition of prostaglandin synthesis, and it is not expected to affect the role of niacin as an NAD precursor. Our patient may have had hypersensitivity to niacin as this therapy is usually associated with flushing as opposed to acute urticarial rash. The escalated dose may have arguably accentuated this side effect. Another relevant question to answer in subsequent studies is whether development of severe dermatological complications maybe a predictor of lack of sustained response to niacin in patients with PEBEL‐1, and whether patients being considered for high‐dose niacin therapy may benefit from prophylactic use of COX inhibition in anticipation of dermatological side effects that may impede tolerance.

In summary, this is the first report of a patient with PEBEL‐1 who showed transient response to niacin but rapidly deteriorated to a fatal outcome despite being started on high dose niacin therapy. It also describes repurposed use of a COX2 inhibitor in the treatment of niacin‐related urticaria in a child with PEBEL‐1. The report argues that patients with PEBEL‐1 and PEBEL‐2 who appear to be improving on niacin might still be at risk of developing catastrophic, life‐threatening encephalopathy. Longitudinal studies and patients' registry maybe needed to answer the questions about long‐term effects and outcomes in patients with PEBEL‐1 treated with niacin. Despite our single observation, authors believe that it is still worth it to try this relatively harmless treatment in an otherwise untreatable condition with potentially fatal outcome.

## AUTHOR CONTRIBUTIONS

Fatema Al‐Amrani and Khalid Al‐Thihli wrote the first draft of the manuscript. Eiman Al‐Ajmi brought her neuroradiology expertise and helped drafting and editing the radiology part. Amna Al‐Futaisi helped revise the neurology part of the manuscript. Fathiya Al‐Murshedi helped conceptualize the idea of the written manuscript and approved the final manuscript as submitted.

## CONFLICT OF INTEREST STATEMENT

Fatema Al‐Amrani, Khalid Al‐Thihli, Eiman Al‐Ajmi, Amna Al‐Futaisi, and Fathiya Al‐Murshedi declare that they have no conflict of interest.

## FUNDING INFORMATION

No funding was received toward this work.

## ETHICS STATEMENT

This study was conducted in agreement with the Declaration of Helsinki and the standard ethical principles governing patients care.

## INFORMED CONSENT

An informed consent for this publication was obtained from both parents of the proband. In addition, all investigations and interventions described the patients were subjected to were part of standard clinical care, and patients were consented appropriately for genetic testing.

## ANIMAL RIGHTS

This article does not contain any studies involving human or animal subjects.

## Data Availability

All details pertaining to the data presented in this manuscript are available for anonymous review.
